# Prevalence of M75 *Streptococcus pyogenes* Strains Harboring *slaA* Gene in Patients Affected by Pediatric Obstructive Sleep Apnea Syndrome in Central Italy

**DOI:** 10.3389/fmicb.2017.00294

**Published:** 2017-02-28

**Authors:** Elisa Viciani, Francesca Montagnani, Giacinta Tordini, Antonio Romano, Lorenzo Salerni, Andrea De Luca, Paolo Ruggiero, Andrea G. O. Manetti

**Affiliations:** ^1^GSK Vaccines S.r.l.Siena, Italy; ^2^Host-Microbiota Interaction Team, Wellcome Trust Sanger InstituteCambridge, UK; ^3^Department of Medical Biotechnologies, University of SienaSiena, Italy; ^4^Hospital Department of Specialized and Internal Medicine, University Division of Infectious DiseasesSiena, Italy; ^5^Clinica Otorinolaringoiatrica, Policlinico Universitario “Le Scotte”Siena, Italy

**Keywords:** *Streptococcus pyogenes*, obstructive sleep apnea syndrome (OSAS), *slaA* gene, Multilocus Sequence Typing (MLST), molecular epidemiology

## Abstract

Recently we reported an association between pediatric obstructive sleep apnea syndrome (OSAS) and Group A *streptococcus* (GAS) sub-acute chronic tonsil colonization. We showed that GAS may contribute to tonsil hyperplasia via a streptolysin O (SLO)-dependent cysteinyl leukotrienes (CysLTs) production, which can trigger T and B cell proliferation. In the present study, we characterized the GAS strains isolated from pediatric OSAS patients in comparison with a panel of age and sex matched GAS strains unrelated to OSAS, but isolated in the same area and during the same period ranging from 2009 to 2013. We found that *slaA* gene, previously reported to be associated to CysLTs production pathway, was significantly associated to GAS OSAS strains. Moreover, the most numerous group (32%) of the GAS OSAS strains belonged to M75 type, and 6 out of 7 of these strains harbored the *slaA* gene. Multilocus Sequence Typing (MLST) experiments demonstrated that the clone emm75/ST49/ *smeZ, slaA* was associated to OSAS cases. In conclusion, we found an association between *slaA* gene and the GAS OSAS strains, and we showed that the clone emm75/ST49 harboring genes *smeZ* and *slaA* was exclusively isolated from patients affected by OSAS, thus suggesting that this genotype might be associated to the pathogenesis of OSAS, although further studies are needed to elucidate the possible role of *SlaA* in tonsil hypertrophy development.

## Introduction

Obstructive sleep apnea syndrome (OSAS) has emerged in children, as the primary indication for surgical removal of adenoids and tonsils (American Thoracic Society, 1996; Marcus et al., 2012; Ramos et al., 2013). OSAS has been associated with cardiovascular, growth and neurobehavioral abnormalities, inflammation, and primarily with hypertrophy of the tonsils and adenoids (Goldbart et al., 2004; Marcus et al., 2012; Zautner, 2012; Kheirandish-Gozal et al., 2013).

*Streptococcus pyogenes* is a human pathogen causing a wide range of diseases (Cunningham, 2000). Although, GAS is considered the most common single organism associated with bacterial pharyngo-tonsillitis (Sidell and Shapiro, 2012), we recently reported an association between pediatric OSAS and GAS tonsil colonization (Viciani et al., 2016). We showed that the GAS toxin SLO was involved in cysteinyl leukotrienes (CysLTs) production, through a SLO-dependent TLR4-mediated, TRIF and MyD88-dependent p38 MAPK pathway. Cysteinyl leukotrienes were able to activate primary T and B cell proliferation *in vitro*, which is possibly related with the development of tonsil hypertrophy, and thus with pediatric OSAS (Viciani et al., 2016).

Following the genome analysis of the M3 GAS strain MGAS315, the prophage-encoded extracellular phospholipase A_2_ (PLA2) SlaA was identified (Beres et al., 2002). SlaA was reported to cleave palmitic and oleic fatty acids, releasing arachidonic acid (Nagiec et al., 2004), as in the case of the cytosolic phospholipase A_2_ (cPLA2), which also releases the arachidonic acid from membrane phospholipids favoring its conversion to leukotrienes and CysLTs by the 5-lipoxygenase pathway (Funk, 2001). This may lead to final tonsil hypertrophy, and thus to OSAS (Viciani et al., 2016). SlaA expression is increased upon contact with cultured epithelial cells and during growth in human saliva (Banks et al., 2003; Shelburne et al., 2005); moreover, individuals with serotype M3 GAS infections produced antibodies against SlaA, indicating that the enzyme is expressed *in vivo* during the course of human diseases (Beres et al., 2002). Of note, the isogenic Δ*slaA* mutant strain was severely compromised in its ability to colonize the upper respiratory tract, leading to the conclusion that SlaA is a key colonization factor (Sitkiewicz et al., 2006).

In this study, we characterized the GAS strains isolated from pediatric OSAS patients, investigating the possible association between a panel of GAS virulence factors (including SlaA) and OSAS. Moreover, we characterized the genotype of the GAS strains isolated from OSAS patients, to investigate whether GAS tonsil colonization could be related to specific clones. To our knowledge, this is the first study that reports an association between emm75/ST49 strains harboring *smeZ* and *slaA* genes and pediatric OSAS.

## Methods

### Study design, patients, and clinical procedures

As recently reported (Viciani et al., 2016), between October 2009 and December 2013, we performed a prospective case-control study on 120 pediatric patients admitted for tonsillectomy to the Otorhinolaryngology Unit of the University Hospital of Siena, which is the only reference unit for this type of surgery in the Siena province and south-east regional area (Tuscany, Central Italy). Overall population of Siena province amounts to 269,388 people among which 36,418 are pediatric subjects (0 to 16 years of age). The University Hospital of Siena is a 700-bed hospital and a total of 900 surgical operations/years are usually performed at the Otorhinolaryngology Unit, admitting patients from Siena city, all areas of the province and from Grosseto and Arezzo provinces. Eligible tonsillectomized patients were clinically stable children (aged ≤ 16 years) affected by OSAS in absence of any signs and symptoms of respiratory diseases, pharyngitis and/or recurrent pharyngitis. Patients with airway obstruction and clinical features of OSAS (e.g. intermittent breathing pauses, heavy snoring, and daytime sleepiness) due to severe palatine tonsil hypertrophy (Paradise et al., 2002), were included in the OSAS group. Clinical diagnosis was confirmed by preoperative evaluation of the patients' medical histories based on specific questions to the parents/caregiver and on physical examinations. Exclusion criteria were: (I) antimicrobial treatment within 10 days before surgery; (II) coexisting chronic cardiac, hepatic, renal or pulmonary diseases; (III) acquired or congenital immunodeficiency; (IV) functional or anatomical asplenia; (V) systemic corticosteroids therapy; (VI) diabetes; (VII) craniofacial syndromes; (VIII) neuromuscular disorders, or (IX) cranial nerve palsies. Among the 120 pediatric subjects admitted for tonsillectomy at the University Hospital, 40 patients were affected by OSAS and in 19 of them we isolated one or more GAS strains, for a total of 22 isolates. In the present study, we matched them with 59 GAS strains isolated from 59 age and sex matched non-OSAS subjects recruited in the same period at the same Hospital of Siena in order to characterize the previously isolated OSAS GAS strains. All strains were community acquired because collected within 12 h from admission. Swabs were performed by different operators immediately before the surgery, during sedation.

### Isolation and characterization of bacterial strains

As previously reported (Viciani et al., 2016), tonsil swabs and cores underwent microbiological analysis. Each specimen was plated on Columbia CNA agar (CNA). Plates were incubated overnight at 37°C in a 5% CO_2_ enriched atmosphere. Overnight cultures were identified on the basis of macro- and microscopic morphology and identity was confirmed through specific biochemical tests, according to standard methods (Murray and Masur, 2012). Glycerol stocks were made for each bacterial strain and stored at −80°C.

### Genomic DNA extraction PCR and sequence analysis

Genomic bacterial DNA employed as template for PCRs was obtained by boiling the bacterial cells, according to the CDC protocol (http://www.cdc.gov/streplab/protocol-emm-type.html). The genomic DNA was immediately used as template for PCRs or stored at −20°C for subsequent studies. In order to amplify the *speA, speB, speC, slo, ssa, sil, smeZ*, and *slaA* genes, primer pairs were designed as described previously (Jing et al., 2006).

Primers SlaAF: 5′-AGTAATAAATACTATTCTATTAGCT-3′, and SlaAR: 5′-TTAACATCCTATAGAACCTACTGT-3′ were used to amplify PCR products and to sequence them using an Applied Biosystems model 3,730 × l DNA Analyzer instrument.

### Co-culture of GAS strains with A549 human lung epithelial cells

A549 cells were cultured on 24-well tissue culture plates with DMEM containing 25 mM Hepes, 0.1% glutamine, 10% fetal bovine serum and antibiotics until they reached 80% confluence. The growth medium was removed, and the cells were washed with phosphate-buffered saline (PBS). One ml of fresh medium without antibiotic was added, and the cells were incubated for 2 h. During this time, overnight cultures of GAS test strains were inoculated into 10 ml of fresh THY medium and cultured to an A_600_ = 0.3. The bacteria were collected by centrifugation, washed once in phosphate-buffered saline, and suspended in 200 microliters of PBS. Hundred microliters of bacteria were added to the A549 cells and incubated for 1 h and 30 min at 37°C. To test the time course of induction, one well was harvested immediately to serve as a 0 h control. After 1 h and 30 min, samples were trypsinized to collect all the bacteria and centrifuged at 14,000 × g for 5 min. Pellets were immediately frozen and stored at −80°C.

### Total RNA isolation, cDNA synthesis, and real-time qPCR

RNA extraction and RT-PCR experiments were performed modifying what previously reported (Viciani et al., 2016). Briefly, total RNA was purified from bacterial pellets co-cultured with A549 cells and collected at time 0 and after 1 h 30′. We performed two 30 s disruptions in 1 ml TRIzol (Invitrogen), using Lysing Matrix B (MP Biomedicals; Solon, OH) in a Fast Prep FP210 Homogenizer (MP Biomedical) with speed setting 6.5. Samples were incubated on ice between each disruption. After 5 min incubation at rt (room temperature), we centrifuged samples at 14,000 × g for 1 min. Total RNA in TRIzol was isolated with Direct-zol RNA miniprep (Zymo research) according to the manufacturer's recommendations, treated with 20 U of Turbo DNase (Ambion), and incubated at 37°C in a thermal bath for 30 min. Samples were then purified and concentrated with RNA clean & concentrator-5 (Zymo research). We estimated RNA concentration with the ND-1000 Spectrophotometer (NanoDrop Technologies, Wilmington, DE, USA). We designed primers for *slaA* amplification (forward: 5′-SlaART_F: TTTAAAGCTAGTTGGCCTGTCC-3′ and reverse: SlaART_R: 5′- ACAATTAGCACCAATACCGGC-3′) from *S. pyogenes* M3, strain GAS315 *slaA* sequence in GenBank (http://www.ncbi.nlm.nih.gov/; NC_004587.1; Gene ID: 1257910) using the software Primer3 (http://simgene.com/Primer3). Previously validated reference *gyrA* gene expression (primers: forward: *gyrA*RT_F: 5′-CGACTTGTCTGAACGCCAAA-3′ and reverse *gyrA*RT_R: 5′-TTATCACGTTCCAAACCAGTCAA-3′) was used as the endogenous control for normalization of the data. Amplification efficiency was established for each of the genes from serial dilutions of *S. pyogenes* genomic DNA. The reactions were performed in a Light Cycler 480 II (Roche) using the Light Cycler RNA amplification kit SYBR green I (Roche) according to the manufacturer's instructions. Each gene was analyzed in triplicate and results were evaluated using Light Cycler® 480 SW 1.5 software (Roche). All reactions amplified a single product as determined by melting curve analysis. The expression of *slaA* gene as compared with the reference gene *gyrA* expression was evaluated with the relative quantification method (ΔΔCT-Method). Data were represented as relative amounts of mRNA normalized to a *gyrA* control.

### Antibiotic susceptibility tests

*In vitro* susceptibility to macrolides and lincosamides was tested in all 81 isolates. Assays were performed and the results were interpreted according to Clinical Laboratory Standards Institute (CLSI) indications (CLSI, 2012). Susceptibility to 14- and 16-membered ring macrolides (erythromycin, spiramycin) and to lincosamides (clindamycin) was determined by the Kirby–Bauer disk diffusion method on Mueller–Hinton agar with 5% sheep blood plates (Oxoid/Thermo Scientific). In addition to the CLSI double disk indication, a triple-disk diffusion test was performed to highlight any possible heterogeneity of inducible resistance, as described previously (Giovanetti et al., 1999).

### *emm* typing

emm typing was carried out according to CDC guidelines (http://www.cdc.gov/streplab/protocol-emm-type.html). Enzymatic extraction of streptococcal genomic DNA was replaced by bacterial boiling in distilled water (Tewodros and Kronvall, 2005) and PCR amplification of the *emm* gene was carried out by the CDC protocol and PCR product sequencing. Sequenced PCR products were then compared with the CDC database to retrieve the *emm* type.

### MLST analysis

Multilocus Sequence Typing (MLST) analysis was conducted on all the M75 strains, as previously described (Enright et al., 2001). This procedure was performed by sequencing seven housekeeping genes (*gki, gtr, murI, mutS, recP, xpt*, and *yqiL*) according to the *S. pyogenes* MLST website (http://pubmlst.org/spyogenes/) sited at the University of Oxford (Jolley and Maiden, 2010). We then compared M75 GAS OSAS strains with M75 GAS strains belonging to three different collections. Collection one contained a miscellaneous of M75 strains isolated from patients affected by a number of different pathologies, and was obtained from the University of Siena, Italy (*n* = 5); collection two contained strains isolated from patients affected by throat or skin infection, and was obtained from the University of Rostock, Germany (*n* = 7); collection three contained strains isolated from patients affected exclusively by pharyngitis, and was obtained from Baylor College of Medicine Houston, Texas, USA (*n* = 16).

### Statistics

**Figure 1:**
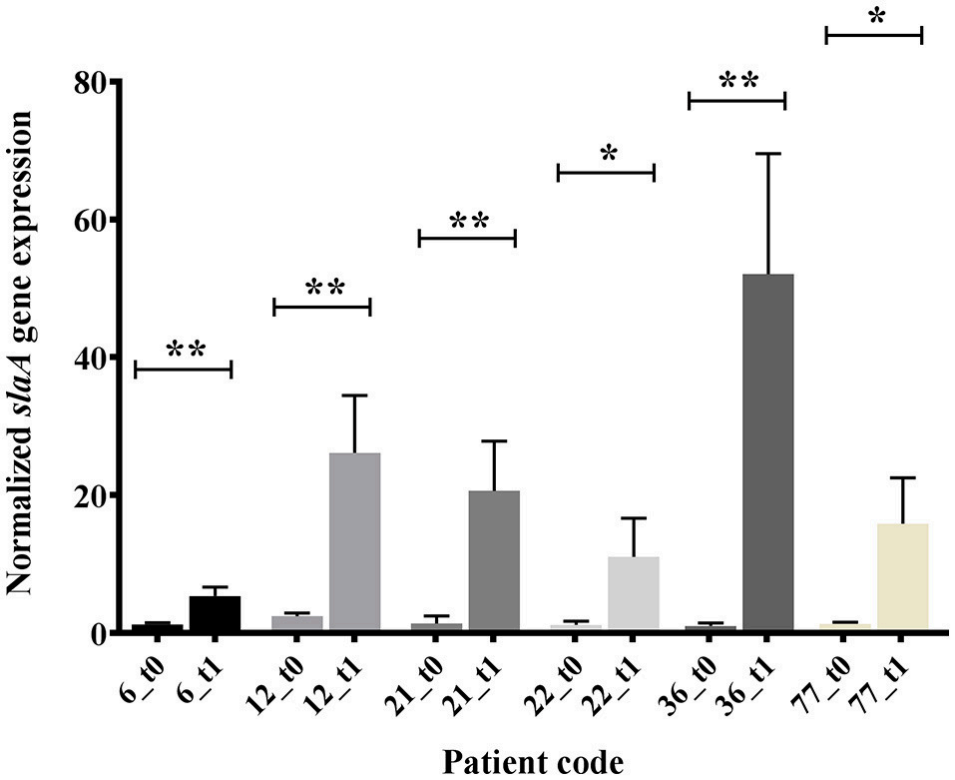
***In vitro*** **expression analysis (real time qRT-PCR), of ***slaA*** in GAS OSAS isolates co-cultured with A549 human epithelial lung cells and collected at time 0 (t0) or after 1 h 30 min (t1)**. The quantity of cDNA for *slaA* gene was normalized to the quantity of *gyrA* cDNA in each RNA sample. The reported values, expressed as fold changes, are the means ± standard errors from three independently isolated RNA preparations analyzed in triplicate. ^*^p < 0.05, ^**^p < 0.01, Unpaired *t*-test.

Non-parametric data are presented as median. Data were analyzed using *Z*-test (Table 1 sex data; Tables 2–**4**), non-parametric Kolmogorov-Smirnov (Table 1 age data), or unpaired *t*-test (Figure 1). For all analyses, *p* < 0.05 was considered significant. Analyses were done with R software and GraphPad Prism 7.02 software.

**Table 1:** **Demographics of the study population stratified by OSAS (obstructive sleep apnoea syndrome), and matched with non-OSAS subjects**.

**Characteristics**	**OSAS**	**Non-OSAS**	***p-*****value**
No. of patients	19	59	–
Age, years	6 (5–9) (4–11)	6 (5–9) (1–11)	0.77
Sex, male	10 (52.6%)	31 (52.5%)	0.99

*Age, median [interquartile range (IQR)] and (minimum age − maximum age). Sex, male numbers (%). Statistical analysis was performed using a non-parametric Kolmogorov-Smirnov test on the distribution of age data, and a Z-test on the proportion of cases and controls for sex data (significance level p = 0.05)*.

**Table 2:** **Distribution of virulence genes among GAS strains isolated from pediatric OSAS patients and matched GAS strains isolated from non-OSAS subjects**.

	***speA***	***speB***	***speC***	***smeZ***	***slo***	***ssa***	***sil***	***slaA***
GAS OSAS (22)	18.1% (4)	100.0%	45.5% (10)	72.7% (16)	100.0%	0.0%	18.2% (4)	45.5% (10)
GAS non-OSAS (59)	25.4% (15)	100.0%	45.7% (27)	74.6% (44)	100.0%	0.0%	18.6% (11)	13.5% (8)
*p*	0.49	1	0.98	0.86	1	1	0.96	0.002

*Statistical analysis was performed using a Z-test on the proportion of cases and controls (significance level p = 0.05). In each cell the percent of isolates is reported, and in brackets the corresponding number of isolates when appropriate*.

### Ethical declarations

This study was carried out in accordance with the recommendations of the Ethical Committee of the Azienda Ospedaliera Universitaria Senese at Siena Hospital with written informed consent from all subjects. All subjects and in particular all the next of kin, caretakers, or guardians on the behalf of the minors/children participants gave written informed consent in accordance with the Declaration of Helsinki. The protocol was approved by the Ethical Committee of the Azienda Ospedaliera Universitaria Senese at Siena Hospital.

## Results

### Presence of *slaA* gene is prevalent in pediatric OSAS patients

During the characterization of the GAS strains isolated from pediatric (≤16 years) OSAS patients (Viciani et al., 2016), we amplified by PCR eight genes encoding virulence factors (Jing et al., 2006) both in the 22 GAS strains isolated from 19 OSAS patients and in 59 GAS strains isolated from 59 age and sex matched non-OSAS subjects recruited in the same period and at the same Hospital of Siena. No significant differences were noted in the demographics of the matched populations (Table 1). As shown in Table 2, *speB*, and *slo* were detected in all the GAS isolates analyzed, confirming to be species-specific, whereas the *ssa* superantigen was not present in OSAS or in non-OSAS isolates. Interestingly, *slaA* gene was found significantly associated (*p* = 0.002) with GAS strains isolated from OSAS patients as compared with GAS strains isolated from non-OSAS subjects, suggesting a possible role of this virulence factor in the development of tonsil hypertrophy. None of the other genes analyzed were found to occur significantly more frequently in the GAS OSAS group vs. the non-OSAS group. No significant difference was found in macrolides and lincosamides chemo-susceptibility patterns of the isolates from OSAS and non-OSAS subjects (Table 3).

**Table 3:** **Macrolides and lincosamides antimicrobial susceptibility of GAS isolates from OSAS patients and from matched non-OSAS subjects**.

	**Erythro**	**Clinda**	**Spira**
**GAS OSAS (22)**			
Susceptible	95.5% (21)	95.5% (21)	95.5% (21)
Intermediate	0.0%	0.0%	0.0%
Resistant	4.5% (1)	4.5% (1)	4.5% (1)
**GAS NON-OSAS (59)**			
Susceptible	93.2% (55)	94.9% (56)	94.9% (56)
Intermediate	0.0%	0.0%	0.0%
Resistant	6.8% (4)	5.1% (3)	5.1% (3)
*p*	0.71	0.92	0.92

*Antibiotics, erythromycin (Erythro), clindamycin (Clinda) and Spiramycin (Spira). Statistical analysis was performed using a Z-test on the proportion of Susceptible and Intermediate/Resistant strains (significance level p = 0.05). The percent of isolates is reported in each cell, along with the corresponding number of isolates in brackets, when appropriate*.

### Presence of *slaA* gene correlates with M75 type

The emm type analysis of the GAS strains isolated from OSAS cases or from non-OSAS subjects showed that most of the isolates (32%, *n* = 7) from the GAS OSAS group belonged to M75 type, whereas only 4 out of 59 (6.8%) of the GAS strains isolated from non-OSAS subjects belonged to this M type (*p* = 0.03). Moreover, no other M type was found to be prevalent in OSAS or non-OSAS group (Table 4). Interestingly, 6 out of the 7 M75 strains isolated from patients affected by OSAS harbored the *slaA* gene (Table S1) which, when sequenced, did not presented any allelic variation (data not shown). Moreover, when all 6 M75 stains were co-cultured with A549 human epithelial lung cells, they increased *slaA* gene expression levels (Figure 1), showing to harbor host epithelial cell-inducible expression of *slaA* gene. We then investigated the presence of *slaA* gene in M75 non-OSAS GAS strains isolated from patients affected by pharyngo-tonsillitis, arthropathy, arthralgia, otitis, skin infection or superinfection during infectious mononucleosis, belonging to three different collections of GAS strains isolated in Italy (*n* = 5), Germany (*n* = 7) and USA (*n* = 16). As shown in Table 5, we obtained four different patterns. Pattern one, harboring genes *smeZ* and *slaA*, was prevalent in strains isolated from OSAS patients in Italy. Pattern two, harboring genes *speA, smeZ*, and *slaA*, was prevalent in strains isolated from patients affected by different pathologies in Italy. Pattern three, harboring gene *smeZ* was prevalent in strains isolated from patients affected by throat infection in Germany. Pattern four, harboring genes *speC* and *smeZ* was prevalent in strains isolated from patients affected by pharyngitis in USA. The genes *speB* and *slo* were not reported as they are species-specific. Interestingly, only 4 out of the 28 non-OSAS GAS strains harbored the *slaA* gene (Table 5). Therefore, our results indicated a significant association (*p* = 0.00018) of *slaA* gene with GAS M75 strains isolated from patients affected by OSAS.

**Table 4:** **Distribution of ***emm*** types among GAS strains isolated from OSAS patients and from matched non-OSAS subjects**.

**emm type**	**OSAS *n*. (%)**	**Non-OSAS *n*. (%)**	***p*****-value**
emm75	7 (31.8)	4 (6.8)	0.003
emm4	3 (13.6)	4 (6.8)	0.32
emm89	3 (13.6)	10 (16.9)	0.36
emm3	2 (9.1)	1 (1.7)	0.11
emm12	2 (9.1)	8 (13.5)	0.58
emm1	1 (4.5)	7 (11.8)	0.32
emm5	1 (4.5)	4 (6.8)	0.71
emm28	1 (4.5)	3 (5.1)	0.92
emm77	1 (4.5)	1 (1.7)	0.45
emm87	1 (4.5)	1 (1.7)	0.45
emm18	0 (0)	3 (5.1)	0.28
emm6	0 (0)	3 (5.1)	0.28
emm11	0 (0)	2 (3.4)	0.38
emm78	0 (0)	1 (1.7)	0.54
emm29	0 (0)	3 (5.1)	3.28
emm61	0 (0)	2 (3.4)	0.38
emm92	0 (0)	1 (1.7)	0.54
emm9	0 (0)	1 (1.7)	0.54
Total	22 (100.0)	59 (100.0)	–

*Statistical analysis was performed using a Z-test on the proportion of cases and controls (significance level p = 0.05)*.

**Table 5:** **Distribution of virulence factors and MLST analysis in a panel of emm75 GAS strains isolated from OSAS or non-OSAS patients**.

**Provenience**	**Disease**	**Strain**	***speA***	***speB***	***speC***	***smeZ***	***slo***	***ssa***	***sil***	***slaA***	**ST**	**emm type**
Siena, Italy	OSAS	6_1									49	emm75
Siena, Italy	OSAS	12_1									49	emm75
Siena, Italy	OSAS	21_1									49	emm75
Siena, Italy	OSAS	22_1									49	emm75
Siena, Italy	OSAS	36_3									49	emm75
Siena, Italy	OSAS	77_1									49	emm75
Rostock, Germany	Skin infection	Ro_110									150	emm75
Siena, Italy	Arthropathy	49A22									49	emm75
Siena, Italy	Arthralgia	49A42									49	emm75
Siena, Italy	Otitis	49B75									49	emm75
Rostock, Germany	Throat infection	Ro_14									150	emm75
Rostock, Germany	Throat infection	Ro_24									150	emm75
Rostock, Germany	Throat infection	Ro_25									150	emm75
Rostock, Germany	Throat infection	Ro_91									150	emm75
Rostock, Germany	Skin infection	Ro_103									150	emm75
Houston, Texas, USA	Pharyngitis	10165									49	emm75
Siena, Italy	Osas	118_1									150	emm75
Siena, Italy	Mononucleosis^*^	49A09									49	emm75
Rostock, Germany	Throat infection	Ro_46									150	emm75
Siena, Italy	Pharyngitis	62_2									49	emm75
Houston, Texas, USA	Pharyngitis	10012									49	emm75
Houston, Texas, USA	Pharyngitis	10020									49	emm75
Houston, Texas, USA	Pharyngitis	10047									49	emm75
Houston, Texas, USA	Pharyngitis	10089									49	emm75
Houston, Texas, USA	Pharyngitis	10106									49	emm75
Houston, Texas, USA	Pharyngitis	10107									49	emm75
Houston, Texas, USA	Pharyngitis	10576									49	emm75
Houston, Texas, USA	Pharyngitis	10586									49	emm75
Houston, Texas, USA	Pharyngitis	20018									49	emm75
Houston, Texas, USA	Pharyngitis	20059									49	emm75
Houston, Texas, USA	Pharyngitis	20671									49	emm75
Houston, Texas, USA	Pharyngitis	30201									49	emm75
Houston, Texas, USA	Pharyngitis	30207									49	emm75
Houston, Texas, USA	Pharyngitis	30603									49	emm75
Houston, Texas, USA	Pharyngitis	32274									49	emm75

* *GAS tonsillar superinfection during mononucleosis. Gray shades indicate the presence of the gene, while white color highlights the absence of the virulence factor*.

### No correlation was found between ST and OSAS cases

Based on this finding, we decided to perform an MLST analysis of M75 strains, in order to investigate whether the pattern harboring *slaA* gene was due to clonality or to different GAS strains. Overall, emm75/ST49 type was isolated in Italy and USA, harboring mostly pattern one, two and four, but also in one case pattern three (Table 5). On the other hand, clone emm75/ST150 was prevalent in pattern three in GAS strains isolated in Germany, harboring neither *slaA* gene nor *speC* gene. However, emm75/ST150 strains were also found harboring pattern one and pattern three (Table 5). As shown in Table 5, the MLST analysis revealed that a single clone was responsible for pattern one in M75 strains isolated from OSAS patients. In fact, the clone emm75/ST49 harboring *slaA* and *smeZ* genes was associated to OSAS in a period ranging from 2009 to 2011 (Table S1); however, when *spe*A gene was additionally present (pattern two) no association with OSAS patients was observed.

### The genotype M75/ST49 harboring *slaA* gene is prevalent in OSAS cases

The characterization of the GAS strains isolated from OSAS patients showed the significant prevalence of *slaA* gene in these isolates as compared with non-OSAS GAS strains isolated in the same area. Furthermore, 6 out of 7 (85.7%) of the M75 GAS strains isolated from OSAS patients harbored *slaA* gene, whereas the analysis for the presence of this gene in a panel of non-OSAS M75 strains, isolated in different countries, showed that only 4 out of 28 strains (14.3%) harbored *slaA* gene. Finally, we showed that all the M75/ST49 GAS strains colonizing OSAS patients harbored genes *smeZ* and *slaA*, a unique clone circulating in a period ranging from 2009 to 2011. In fact, interestingly, this GAS clone was never isolated from non-OSAS patients examined in the same area and in the same period. Of note, unlike *slaA*, the superantigene *smeZ* was not prevalently associated with GAS strains isolated from OSAS patients when compared with GAS strains isolate from non-OSAS subjects (*p* = 0.86, Table 2), suggesting that this virulence factor is not particularly associated to patients affected by OSAS. It has also to be noted that in M75/ST49 strains the presence of *speC* gene is negatively associated to OSAS cases (*p* = 0.0003).

Taken together these data show the association of GAS clone emm75/ST49 harboring *slaA* gene with pediatric patients affected by OSAS, leading to hypothesize a possible role of the SlaA protein in the pathogenesis of tonsil hypertrophy, and thus possibly of obstructive sleep apnea syndrome.

## Discussion

We recently reported an association between pediatric OSAS and GAS tonsil colonization (Viciani et al., 2016). Based on our data, we hypothesized a mechanism of pathogenesis where tonsil hypertrophy, the main risk factor for pediatric OSAS, could be determined by the proliferative effect that the GAS toxin SLO-dependent TLR4-mediated, TRIF and MyD88-dependent CysLTs production has on tonsil T and B cells (Viciani et al., 2016). Here, we found that *slaA* gene, previously reported to be involved in leukotrienes production pathway (Funk, 2001), is significantly associated to GAS OSAS strains and in particular to the clone emm75/ST49 harboring *smeZ* and *slaA* genes.

In 1997, erythromycin resistance was reported to be in 53% of GAS isolates from Siena; 10 years later the same resistance phenotype decreased to 16%, these phenotypes being equally distributed between M (14 and 15 ring member macrolide-resistant) and MLS (all macrolide, lincosamide, and streptogramin-resistant) (Cresti et al., 2002; Montagnani et al., 2009). Although, the genotype emm75/ST49 was one of the most frequent M-phenotype, carrying *mef(A)* gene, isolated during surveys conducted in USA, Spain, Korea and Japan (Green et al., 2006; Ardanuy et al., 2010; Takahashi et al., 2016), in our study we found 1 MLS resistant phenotype in all the GAS strains isolated from the OSAS patients, and 3 MLS resistant phenotypes plus 1 M phenotype in strains isolated from matched non-OSAS group, no one belonging to M75 type. This very low macrolide resistance rate was further confirmed by a recent study conducted in the same area (Olivieri et al., 2015). In a study conducted in Portugal between 2,000 and 2,005, the genotype emm75/ST150 was significantly associated with pharyngitis (Friaes et al., 2012); however, interestingly, a mucoid form of a GAS strain M75/ST49 has recently been reported to cause a severe acute otitis media (Kakuta et al., 2014).

The analysis of the presence of eight virulence factors in the genome of the GAS OSAS strains showed the species-specificity of *speB* and *slo* as previously reported (Jing et al., 2006; Luca-Harari et al., 2009). Moreover, the superantigen gene *ssa* was not found in our isolates, but was present in 31% of the strains isolated in a European survey (Luca-Harari et al., 2009), and in 23% of the isolates in a Chinese study (Jing et al., 2006). The superantigen gene *smeZ* was highly represented in our isolates, as described in other surveys (Jing et al., 2006; Luca-Harari et al., 2009). In agreement with previous data, *spe*A was associated with M1 and M3, often involved in severe infections with high mortality, whereas *speC* was isolated in several other types, also associated with high mortality (Schmitz et al., 2003; Jing et al., 2006; Luca-Harari et al., 2009). The M1 and M3 strains have the gene *speA* and lack gene *speC*, suggesting that the presence of *speA* affects the acquisition of *speC* (Schmitz et al., 2003). In China *speA* was more prevalent in invasive than in epipharynx isolates, while *speC* was significantly over-represented in epipharynx isolates (Jing et al., 2006). Another gene related to invasive GAS strains is *sil* (Hidalgo-Grass et al., 2004), which we found in 18.2% of OSAS strains similarly to GAS isolated from China (Jing et al., 2006).

Following the genome analysis of the M3 GAS strain MGAS315, a prophage-encoded extracellular phospholipase A_2_ (PLA2) named SlaA involved in the production of pro-inflammatory lipid mediators such as leukotrienes was identified. SlaA had not been present in M3 GAS strains before 1987, when a resurgence of severe invasive disease episodes occurred (Beres et al., 2002; Ikebe et al., 2002). These data, together with the reported lack of allelic variation, suggest that the *slaA* gene was recently acquired by transduction by a distinct serotype M3 sub-clone that then spread globally (Nagiec et al., 2004; Sitkiewicz et al., 2007). It has been reported that *slaA* was present, even if uncommonly, in few other serotypes, including M1, M2, M4, M6, M22, M28, and M75 strains (Nagiec et al., 2004).

Of note, *slaA* gene was found in 45.5% of the GAS strains isolated from OSAS patients, whereas the occurrence rate of this gene has been reported to be much lower (10.8% in Europe, USA or Canada and 4.65% in China) (Nagiec et al., 2004; Jing et al., 2006). On this regard, GAS M75 strains harboring *slaA* gene, isolated in 85.7% of the OSAS subjects colonized by GAS, were reported to be uncommon (Nagiec et al., 2004; Jing et al., 2006; Kittang et al., 2011) and, when occurring, associated with invasive strains (Nagiec et al., 2004). Recently, it has been described a high expression of *slaA* and of the adjacent gene *speK* in a skin-tropic invasive GAS strain, implying an involvement of SlaA in host-pathogen interactions in the skin and a possible role in enhancing virulence and fitness in adaption to host niches (Bao et al., 2016).

It has been shown that *slaA* expression was induced upon contact with cultured human pharyngeal epithelial cells (confirmed in this study) and during growth in human saliva (Banks et al., 2003; Shelburne et al., 2005), and that individuals with serotype M3 GAS infections seroconvert to SlaA, indicating that the enzyme is expressed *in vivo* during the course of human diseases (Beres et al., 2002). Moreover, interestingly, in a cynomolgus macaque model of pharyngitis (Sumby et al., 2005; Virtaneva et al., 2005), the isogenic *slaA* mutant strain was severely compromised in its ability to colonize the upper respiratory tract, leading to the conclusion that SlaA is a key colonization factor (Sitkiewicz et al., 2007). It has been demonstrated that SlaA, which is involved in the generation of pro-inflammatory lipid mediators such as leukotrienes (Nagiec et al., 2004), is regulated by TLR4 signaling in LPS-activated macrophages and that the regulation occurs through TLR4-mediated MyD88- and TRIF-dependent MAPK signaling pathways (Qi and Shelhamer, 2005). In this study, we observed a significant association between OSAS cases and GAS strains genotype emm75/ST49 harboring *slaA*,. Given that SlaA can induce CysLTs production and that the latter are involved in tonsillar T and B cell proliferation, these results could enforce the recent discovery of a possible role of GAS virulence factors in tonsil hypertrophy and pediatric OSAS development (Viciani et al., 2016). However the found correlation does not imply causation; the multifactorial nature of the disease and the fact that our data refer to a limited number of cases and are from a single center in central Italy suggest that the study sample might not be representative of the whole Italian population. Ideally our studies should be replicated in a large independent, multicenter cohort. Moreover, since only a subset (45.5%) of GAS OSAS strains in our study harbors *slaA* gene, we cannot rule out the importance of other risk factors, such as SLO toxin; thus *slaA* gene should be considered as a part of a combination of factors, which may have a possible role in chronic diseases.

## Author contributions

Conceived and designed the experiments, reviewed and approved the final version of the manuscript and analyzed the data: EV, FM, GT, AR, LS, AD, PR, and AM. Performed the experiments: EV, GT, and AM. Wrote the paper: EV, FM, PR, and AM.

### Conflict of interest statement

AM and PR were employees of Novartis Vaccines and Diagnostics Srl at the time of the study. Following the acquisition of Novartis Vaccines by the GSK group of companies in March, 2015, AM is now employee of the GSK group of companies, while PR retired. EV was a PhD Student of the University of Siena at the time of the study and supervised by Novartis Vaccines and Diagnostics Srl. EV has now a postdoctoral position at Wellcome Trust Sanger Institute, UK. AD has a personal fee from Merck Sharp and Dohme. AD, FM, AR, and LS have done contract research for Novartis Vaccine and Diagnostics S.r.l. (now acquired by the GSK group of companies) on behalf of the Hospital University of Siena, Italy. This work was sponsored by Novartis Vaccines and Diagnostics Srl, now acquired by the GSK group of companies, who were involved in all stages of the study conduct and analysis. GSK took responsibility for all costs incurred in publishing. The other authors declare that the research was conducted in the absence of any commercial or financial relationships that could be construed as a potential conflict of interest.
