# Supplementary material for: Prevalence of M75 Streptococcus pyogenes Strains Harboring slaA Gene in Patients Affected by Pediatric Obstructive Sleep Apnea Syndrome in Central Italy
Source: Front Microbiol. 2017 Feb 28;8:294. doi: 10.3389/fmicb.2017.00294 (PMC5329643; doi:10.3389/fmicb.2017.00294)
Supplement: Supplementary file 1 [file Table1.DOCX]

**Supplemental Materials**

**Table S1**

| **Date of isolation** | **Provenience** | **Disease** | **Strains** | ***speA*** | ***speB*** | ***speC*** | ***smeZ*** | ***slo*** | ***ssa*** | ***sil*** | ***slaA*** | **emm type** |
| --- | --- | --- | --- | --- | --- | --- | --- | --- | --- | --- | --- | --- |
| 11_11_2009 | Siena, Italy | OSAS | **2_1** |  |  |  |  |  |  |  |  | **12** |
| 19_11_2009 | Siena, Italy | OSAS | **6_1** |  |  |  |  |  |  |  |  | **75** |
| 17_12_2009 | Siena, Italy | OSAS | **7_1** |  |  |  |  |  |  |  |  | **87** |
| 25_02_2010 | Siena, Italy | OSAS | **12_1** |  |  |  |  |  |  |  |  | **75** |
| 5_05_2010 | Siena, Italy | OSAS | **21_1** |  |  |  |  |  |  |  |  | **75** |
| 10_05_2010 | Siena, Italy | OSAS | **22_1** |  |  |  |  |  |  |  |  | **75** |
| 20_05_2010 | Siena, Italy | OSAS | **23_9** |  |  |  |  |  |  |  |  | **4** |
| 20_05_2010 | Siena, Italy | OSAS | **23_11** |  |  |  |  |  |  |  |  | **89** |
| 23_09_2010 | Siena, Italy | OSAS | **36_3** |  |  |  |  |  |  |  |  | **75** |
| 27_01_2011 | Siena, Italy | OSAS | **61_4** |  |  |  |  |  |  |  |  | **4** |
| 18_02_2011 | Siena, Italy | OSAS | **68_1** |  |  |  |  |  |  |  |  | **3** |
| 24_03_2011 | Siena, Italy | OSAS | **73_1** |  |  |  |  |  |  |  |  | **89** |
| 31_03_2011 | Siena, Italy | OSAS | **77_1** |  |  |  |  |  |  |  |  | **75** |
| 14_04_2011 | Siena, Italy | OSAS | **78_2** |  |  |  |  |  |  |  |  | **12** |
| 16_06_2011 | Siena, Italy | OSAS | **92_1** |  |  |  |  |  |  |  |  | **89** |
| 16_06_2011 | Siena, Italy | OSAS | **92_3** |  |  |  |  |  |  |  |  | **1** |
| 26_01_2012 | Siena, Italy | OSAS | **103_3** |  |  |  |  |  |  |  |  | **4** |
| 31_05_2012 | Siena, Italy | OSAS | **117_1** |  |  |  |  |  |  |  |  | **77** |
| 31_05_2012 | Siena, Italy | OSAS | **118_1** |  |  |  |  |  |  |  |  | **75** |
| 07_06_2012 | Siena, Italy | OSAS | **119_1** |  |  |  |  |  |  |  |  | **5** |
| 06_06_2013 | Siena, Italy | OSAS | **147_1** |  |  |  |  |  |  |  |  | **3** |
| 06_06_2013 | Siena, Italy | OSAS | **147_3** |  |  |  |  |  |  |  |  | **28** |

**Table S1.** Epidemiological data and distribution of virulence factors among GAS strains isolated from OSAS patients.
